# Drugs of Last Resort? The Use of Polymyxins and Tigecycline at US Veterans Affairs Medical Centers, 2005–2010

**DOI:** 10.1371/journal.pone.0036649

**Published:** 2012-05-16

**Authors:** Benedikt Huttner, Makoto Jones, Michael A. Rubin, Melinda M. Neuhauser, Adi Gundlapalli, Matthew Samore

**Affiliations:** 1 VA Salt Lake City Health Care System and University of Utah, Salt Lake City, Utah, United States of America; 2 Department of Veterans Affairs Pharmacy Benefit Management Services, Hines, Illinois, United States of America; Los Angeles Biomedical Research Institute, United States of America

## Abstract

Multidrug-resistant (MDR) and carbapenem-resistant (CR) Gram-negative pathogens are becoming increasingly prevalent around the globe. Polymyxins and tigecycline are among the few antibiotics available to treat infections with these bacteria but little is known about the frequency of their use. We therefore aimed to estimate the parenteral use of these two drugs in Veterans Affairs medical centers (VAMCs) and to describe the pathogens associated with their administration. For this purpose we retrospectively analyzed barcode medication administration data of parenteral administrations of polymyxins and tigecycline in 127 acute-care VAMCs between October 2005 and September 2010. Overall, polymyxin and tigecycline use were relatively low at 0.8 days of therapy (DOT)/1000 patient days (PD) and 1.6 DOT/1000PD, respectively. Use varied widely across facilities, but increased overall during the study period. Eight facilities accounted for three-quarters of all polymyxin use. The same statistic for tigecycline use was twenty-six VAMCs. There were 1,081 MDR or CR isolates during 747 hospitalizations associated with polymyxin use (1.4/hospitalization). For tigecycline these number were slightly lower: 671 MDR or CR isolates during 500 hospitalizations (1.3/hospitalization) (p = 0.06). An ecological correlation between the two antibiotics and combined CR and MDR Gram-negative isolates per 1000PD during the study period was also observed (Pearson’s correlation coefficient r = 0.55 polymyxin, r = 0.19 tigecycline). In summary, while polymyxin and tigecycline use is low in most VAMCs, there has been an increase over the study period. Polymyxin use in particular is associated with the presence of MDR Gram-negative pathogens and may be useful as a surveillance measure in the future.

## Introduction

In recent years, several studies have reported encouraging trends for multidrug-resistant (MDR) Gram-positive pathogens. In one such study, a decrease in health care-associated methicillin-resistant *Staphylococcus aureus* (MRSA) infection rates was observed after the implementation of a MRSA bundle in all Veterans Affairs medical centers (VAMCs) in 2007. [1] Similar trends have been reported from other settings in the United States and from other countries, such as the United Kingdom and France. [2], [3], [4] Unfortunately, these advances have been partly offset by the fact that a series of resistance genes coding for broad-spectrum beta-lactamases–NDM-1, KPC, VIM, OXA-48, CTX-M-15, and AmpC, to name just a few–are increasingly found in Gram-negative bacteria across the globe. [5], [6]

These genes coding for enzymes capable of conveying resistance to broad-spectrum cephalosporins and carbapenems are not only harbored by typical nosocomial pathogens, but also by pathogens associated with the community, such as *Escherichia coli*. [7] The presence of extended-spectrum beta-lactamase (ESBL) genes in the strain of *E. coli* that caused a recent outbreak of hemolytic-uremic syndrome in Germany is a reminder that antimicrobial resistance in Enterobacteriaceae is not a problem limited to hospitals. [8] In addition, multiple resistance genes are often clustered on plasmids, rendering their owners extensively or even pan-drug resistant. [9], [10] Carbapenem resistance is of particular concern since few antibiotics are available to treat infections with carbapenem-resistant (CR) organisms. [11]

This emerging resistance has led to a focus on antibiotics from alternative classes. Tigecycline, an antibiotic structurally related to tetracyclines, was approved in the United States in 2005 and shows activity against MDR Gram-negative pathogens, with a notable gap in coverage of *Pseudomonas* species. [12] Recent randomized controlled trial data showing higher all-cause mortality in tigecycline-treated patients has, however, dampened enthusiasm for this drug. [13], [14], [15] Increasing attention is being paid to the polymyxins, a class of antibiotics that had fallen out of favor with clinicians due to concerns about toxicity and uncertainty about optimal dosing strategies. [16], [17], [18] Despite the fact that the use of polymyxins or tigecycline probably indicates the presence or suspicion of problematic pathogens, there are no comprehensive data about the use of these antibiotics, nor which pathogens motivate their use. The availability of comprehensive antimicrobial prescribing and microbiology data from VAMCs offers a unique opportunity to fill this gap in understanding both an older and newer antimicrobial agent.

## Methods

There are 152 VAMCs providing acute and long-term care across the United States. To capture inpatient data, we included records from acute medical, surgical, neurological, and intensive-care units in our antibiotic and microbiologic analyses. We further restricted the cohort to facilities with at least 10 operational acute-care beds during fiscal years (FY) (October, 1- September, 30) 2006 through 2010, and those with barcode medication administration (BCMA) data available over the entire study period.

### Antibiotic Use Data

We used nationwide, individual-level data drawn from the VA Informatics and Computing Infrastructure (VINCI) for this study. BCMA technology was introduced VA-wide in 2000 to improve medication safety. [19] With each administered dose of medication, data regarding drug and route of administration are recorded electronically with a time stamp. To detect missing data, we assessed the completeness of the BCMA data set by comparing it with electronic orders. We deemed antibiotic BCMA data to be complete in a facility from the first month in which the difference between antibiotic use in BCMA data and standard electronic orders was less than 20% (based on at least one recorded order or BCMA entry) for five commonly used index antibiotics (ciprofloxacin, vancomycin, piperacillin/tazobactam, metronidazole, and ceftriaxone). We used this cutoff because some medication orders are written and cancelled before a single dose can be administered. In addition it should be noted that a bar code quality program to ensure 100 percent scan success rates has been implemented VA-wide in 2006. BCMA data were used to assess the use of parenteral polymyxins (polymyxin B and colistin/colistimethate, which we will refer to collectively as “colistin”) and tigecycline. [9], [10]

Antibiotic use was expressed in terms of “days of therapy” (DOT). One DOT was defined as the administration of a single antibiotic on a given day independent of the number of doses, the strength of the dose administered, or the route of administration. [20] DOT were denominated by patient days using a midnight census approach. As such, the discharge day was not counted in the numerator or the denominator. We also denominated antibiotic use by “hospitalizations” where at least one hospital inpatient day fell within the study period.

### Microbiology Data

The methods used to extract microbiology data from the VA network have been previously described elsewhere with regard to MRSA. [21] We used analogous methods to extract microbiology data for Gram-negative pathogens. In brief, organisms and susceptibilities were extracted into a relational structure from semi-structured text documents using natural language processing (NLP). All inpatient microbiology data between the 7th day before and 1st day after the first day of polymyxin or tigecycline use were also manually reviewed. Only the first unique isolate of a species (defined by the susceptibility pattern) for each hospitalization was analyzed. Isolates without at least one reported susceptibility and cultures obtained in the outpatient setting were excluded. Multi-drug resistance was defined as “acquired” non-susceptibility to at least one agent in three or more antimicrobial categories according to the definitions proposed by a joint initiative by the European Centre for Disease Prevention and Control (ECDC) and the Centers for Disease Control and Prevention (CDC). [22] Carbapenem-resistance definitions were also pathogen-specific and drawn from the same proposal. We were not able to apply the proposed definitions for extensively drug-resistant (XDR) and pan-drug resistant (PDR) bacteria due to the lack of standardization of reported susceptibilities; however, we modified the definition of XDR to be resistance in all reported drug classes except polymyxins. Analyses of multi-drug resistance were restricted to Enterobacteriaceae, *Acinetobacter,* and *Pseudomonas* species due to the lack of comprehensive definitions for other Gram-negative organisms.

We used descriptive statistics to describe antibiotic use and microbiology data. All analyses were performed using STATA version 11 (StataCorp. 2009. College Station, TX).

### Ethics Statement

This study was approved by the Research Review Committee of the VA Salt Lake City Health Care System and Institutional Review Board of the University of Utah. As the study involved retrospective review of existing medical record data with no patient contact, the IRB approved and granted waiver of informed consent to access medical records. All results are presented in aggregate to protect the privacy and confidentiality of study subjects.

## Results

Of 152 VAMCs, 20 did not meet our acute-care services criteria, 1 was excluded for less than 10 acute-care beds, and 4 were excluded for incomplete BCMA data. The remaining 127 VAMCs logged 2.42 million unique hospitalizations on the included wards over the study period accounting for over 13.57 million patient days (table 1).

**Table 1:** Characteristics of the 127 hospitals October 2005 through September 2010.

Hospital Characteristics	Count (%)
**Hospitals with ICUs**	**121 (95.3)**
**University affiliated**	**115 (90.6)**
VA region	
** 1 West**	24 (18.9%)
** 2 Central**	36 (28.4%)
** 3 Southeast**	40 (31.5%)
** 4 Northeast**	27 (21.3%)
	**Value [IQR]**
Average daily occupied acute Care Beds	53.0 [26.3–81.1]
Average daily occupied ICU Beds	8.0 [3.4–13.9]

### Antibiotic Use

There were 11,535 days of therapy (DOT) of polymyxins (0.1% of overall DOT for systemic antibiotics) administered during 1,145 hospitalizations over the study period. Polymyxin B, which is part of the national VA formulary, accounted for 63.7% (7,343 DOT) of all IV polymyxin DOT (the remaining DOT were colistin). In comparison, there were 21,886 DOT of tigecycline during 3,125 hospitalizations.

**Figure 1:**
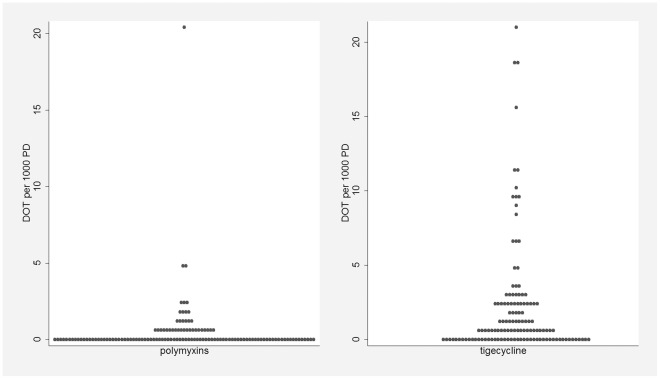
Trends in the use of polymyxin and tigecycline in days of therapy (DOT) per 1000 patient days (PD) by fiscal year.

Tigecycline DOT rose more steeply than polymyxin DOT during the five-year period of analysis (figure 1). From FY 2006, the first full fiscal year of tigecycline approval, to FY 2010, tigecycline use increased 4.2 fold. Polymyxin use peaked in FY 2009 then declined modestly in FY 2010.

**Figure 2:**
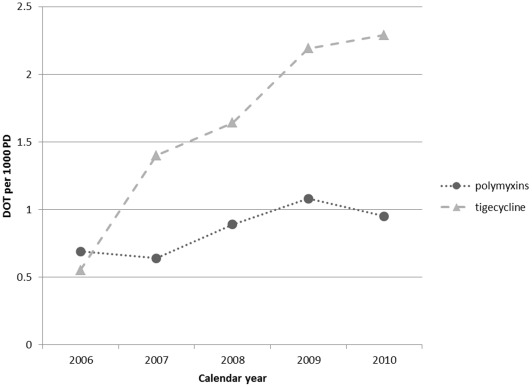
Dot plot of tigecycline and polymyxin use by facility in DOT per 1000 PD in fiscal year 2010. Each dot represents one facility (n = 127).

The distribution of hospital polymyxin use across facilities was more skewed than that of tigecycline use (figure 2). The facility with the highest polymyxin use prescribed 53.9% of all polymyxin DOT. Only 8 (6%) facilities accounted for 75% of polymyxin use. In FY 2010, polymyxin DOT in the highest use facility were more than four times greater than the polymyxin DOT in second highest use facility (20.6 vs 4.9 DOT per 1000 PD). Overall, 54 hospitals used no polymyxins during the five year time frame. In contrast, the facility with the highest tigecyline use accounted for 8.8% of tigecycline DOT. Twenty-six facilities (20%) accounted for 75% of tigecycline DOT over the study period, while 22 hospitals did not use tigecycline at all.

Polymyxin and tigecycline use varied across different geographic regions. Yet, each region demonstrated an upward temporal trend in polymyxin and tigecycline utilization (with the exception of the Northeast where polymyxin prescribing was relatively stable). The comparison of geographic regions was dominated by the effect of an outlier effect. Excluding the highest use facility, overall polymyxin prescribing was 0.7 DOT per 1000 PD in the northeastern region compared to 0.3, 0.3, and 0.4 DOT per 1000 PD in the other three regions. Tigecycline use (again excluding the outlier facility) was also highest in the Northeast at 2.9 DOT per 1000 PD.

Polymyxins were administered for a median of 7 days (IQR 4–13, mean 10.1) and tigecycline for a median of 5 days (IQR 3–9, mean 7.0) per hospitalization. In comparison, the individual median number of days for any antibiotic therapy was 3 days (IQR 2–6) per hospitalization. 49.0% of all polymyxin DOT were administered in intensive care units, while this was the case for only 33.5% of tigecycline DOT.

While 86.8% of all antibiotic courses were started within the first 2 days of admission, polymyxins were started a median of 15 days after the day admission (IQR 4–31) and only 14.8% of polymyxin courses were started within the first 2 days. Tigecycline was started earlier at a median of 4 days after the day admission (IQR 1–12), and 37.1% of all courses were started within 2 days. Patients receiving polymyxins received a median of 12 days (IQR 3–26) of antibiotic therapy before the first dose of polymyxins, while patients receiving tigecycline had received a median of 3 days of antibiotics (IQR 0–10).

Polymyxins were administered mostly in combination with other antibiotics (IV polymyxins were administered alone on only 16.3% of days). On average, 1.6 (SD 1.1) other antibiotics were given concomitantly. Tigecycline was administered with polymyxins on 7.1% of polymyxins days. For comparison tigecycline was given with an average of 0.9 (SD 1.0) other antibiotics and tigecycline was given alone on 44.5% of all tigecycline days.

### Microbiology

**Figure 3:**
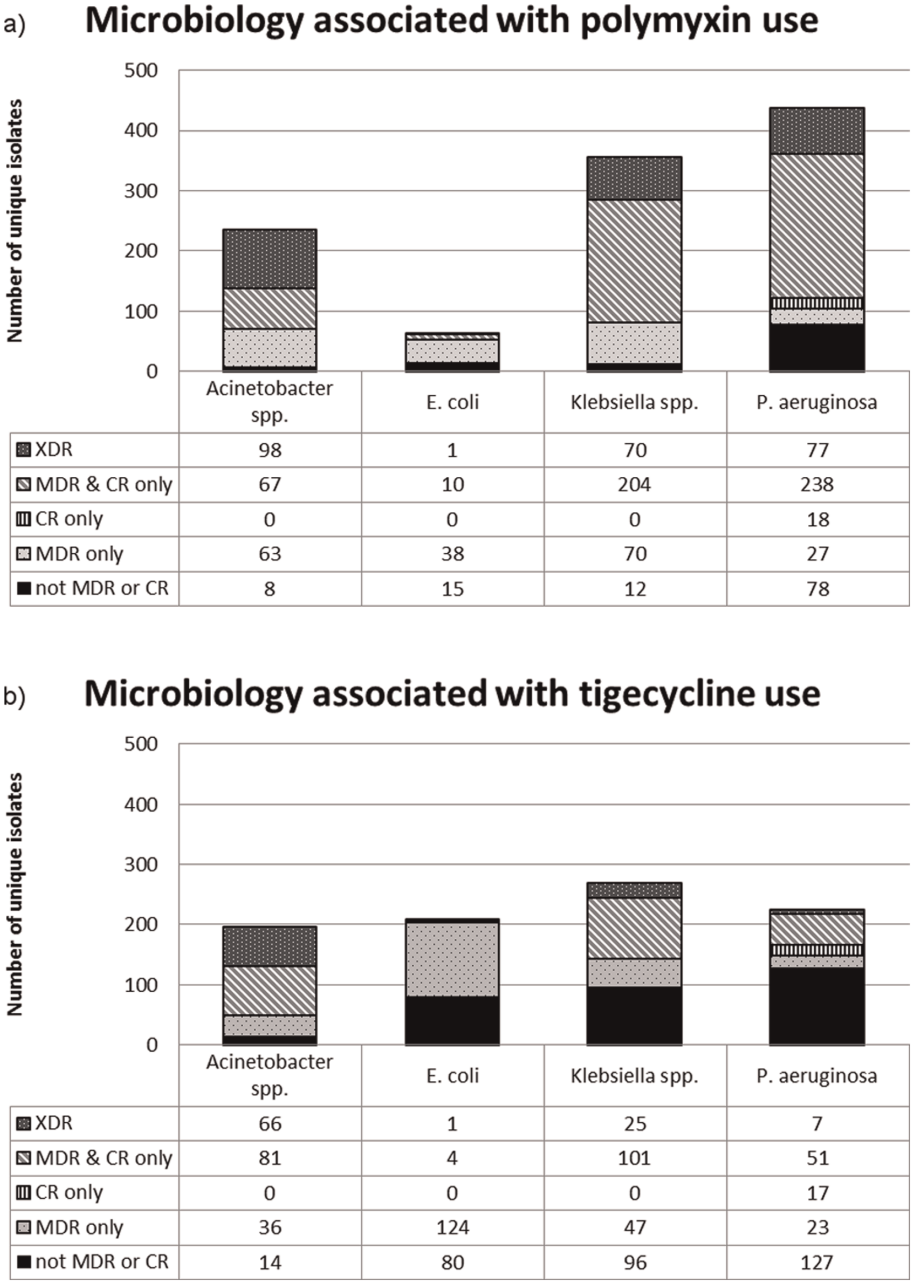
Pathogens isolated. a) when polymyxins (1,145 hospitalizations) or b) tigecycline (3,125 hospitalizations) were used. Only *Acinetobacter spp.*, *E. coli*, *Klebsiella spp.* and P. aeruginosa* are included **MDR:** multi-drug resistance (non-susceptibility to > = 3 drug classes) **CR:** carbapenem resistance (non-susceptibility to carbapenems; ertapenem not considered for non-fermenters) **XDR:** non-susceptibility to all tested classes except to polymyxins (definitions of MDR, CR and XDR adapted from reference [22]) *In 206/225 cases where tigecycline was given and a Pseudomonas was isolated, the patient had at least one anti-pseudomonal spectrum agent (definition of anti-pseudomonal activity based on reference [22]).

There were 1,274 unique Gram-negative isolates recovered between the 7th day before and the 2nd day after the first dose of IV polymyxin each hospitalization. Of these, 1,081 isolates fulfilled criteria for MDR or CR Enterobacteriaceae, *Pseudomonas,* or *Acinetobacter* (figure 3). Among the rest there were 17 *Stenotrophomonas* isolates. The resistant isolates were found among 747 unique hospitalizations (65.2%) when polymyxins were used; no pathogen was identified in 350 hospitalizations (30.6%) (the remainder demonstrating pathogens not fulfilling MDR or CR criteria). *Pseudomonas aeruginosa* (361; 33.4%), *Klebsiella* spp. (344; 31.8%), *Acinetobacter* spp. (228; 21.1%), and *Escherichia coli* (49; 4.5%) together accounted for most resistant isolates. Enterobacteriaceae other than the organisms listed above made up an additional 9.2%. The most common culture site among resistant organisms was sputum (table 2).

Using the same definitions as for polymyxins, there were 1,280 unique Gram-negative isolates for tigecycline (174 of which were also found with concurrent polymyxin use). Of these, 671 met criteria for MDR or CR Enterobacteriaceae, *Pseudomonas*, or *Acinetobacter* (figure 3). There were 70 *Stenotrophomonas* isolates. All of the resistant isolates were found in 500 hospitalizations (16% of all hospitalizations when tigecycline was used). There were 2,281 hospitalizations without pathogens. The remainder were non-resistant isolates.

**Table 2:** Culture sample types by resistant (MDR, CR or XDR) pathogen.

POLYMYXINS				
Culture site	*Acinetobacter spp.*# isolates (%)	*E. coli* # isolates (%)	*Klebsiella spp.* # isolates (%)	*P. aeruginosa* # isolates (%)
**Sputum**	**122 (53.5%)**	**13 (26.5%)**	**138 (40.1%)**	**201 (55.7%)**
**Urine**	**17 (7.5%)**	**13 (26.5%)**	**84 (24.4%)**	**73 (20.2%)**
**Blood**	**55 (24.1%)**	**7 (14.3%)**	**74 (21.5%)**	**38 (10.5%)**
**Other**	**34 (14.9%)**	**16 (32.7%)**	**48 (14.0%)**	**49 (13.6%)**
**TIGECYCLINE**				
**Culture site**	***Acinetobacter spp.*** ** # isolates (%)**	***E. coli*** ** # isolates (%)**	***Klebsiella spp.*** ** # isolates (%)**	***P. aeruginosa*** ** # isolates (%)**
**Sputum**	**93 (50.8%)**	**29 (22.5%)**	**54 (31.2%)**	**49 (50.0%)**
**Other**	**39 (21.3%)**	**64 (49.6%)**	**38 (22.0%)**	**24 (24.5%)**
**Blood**	**35 (19.1%)**	**12 (9.3%)**	**32 (18.5%)**	**10 (10.2%)**
**Urine**	**16 (8.7%)**	**24 (18.6%)**	**49 (28.3%)**	**15 (15.3%)**

Only isolates recovered between the 7th day before and the 1st day after the first day of polymyxin or tigecycline use were included.

**Figure 4:**
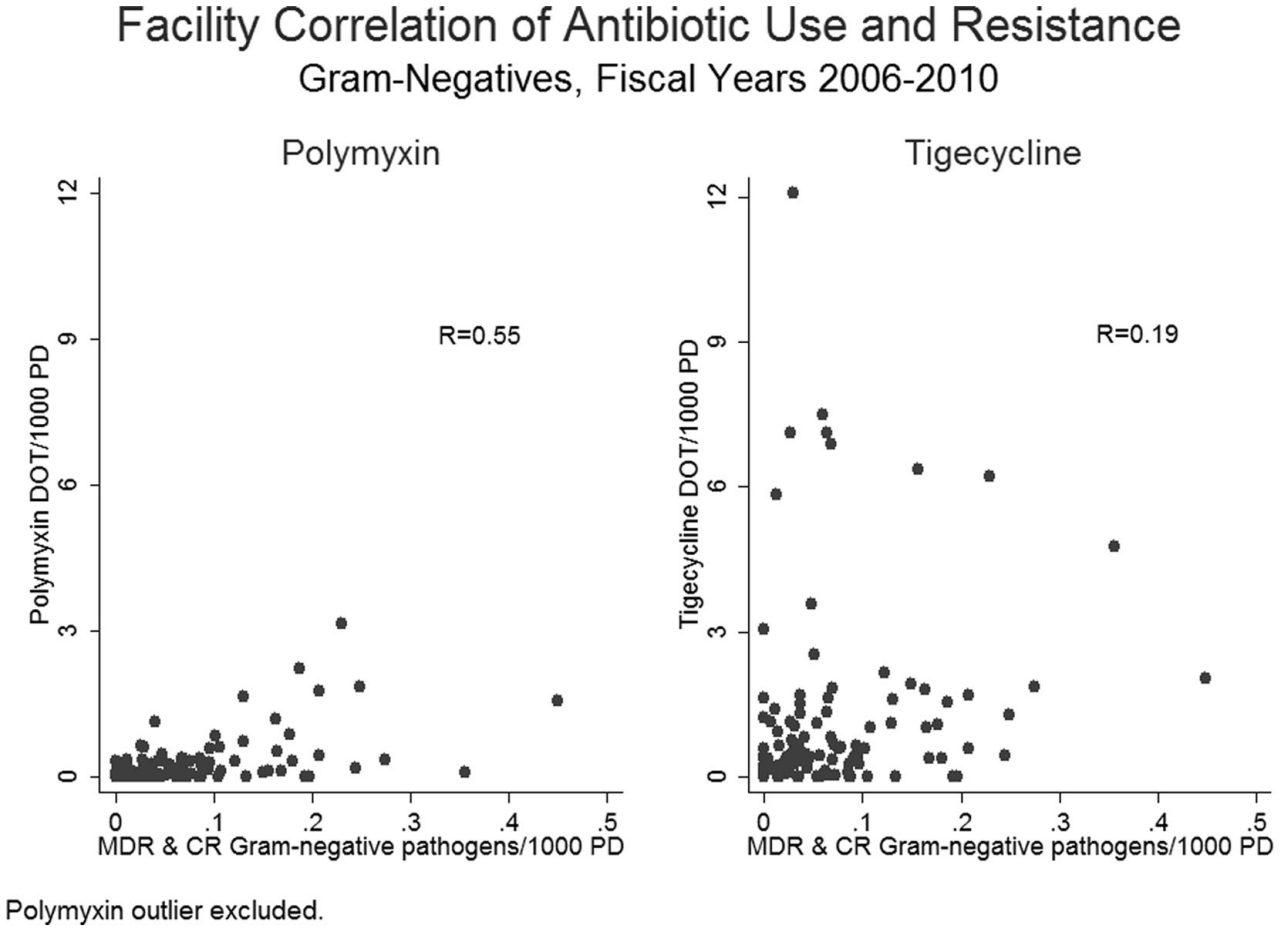
Correlation between polymyxin/tigecycline use in days of therapy (DOT) per 1000 patient days (PD) and MDR & CR Gram-negative pathogens per 1000 PD (aggregated over the entire study period). Extreme polymyxin use outlier excluded.

We also examined the ecological correlation between the two antibiotics and combined CR and MDR Gram-negative isolates per 1000 PD during the time period. Removing the extreme outlier facility, the Pearson correlation coefficient was 0.55 for polymyxin and 0.19 for tigecycline (figure 4).

## Discussion

In this explorative study, we found 1) an increase in the use of “last-resort” antibiotics over the study period, 2) important variation in the use of these antibiotics among facilities, and 3) as expected, the frequent presence of MDR and/or CR Gram-negative pathogens in patients treated with polymyxins.

We observed an increase in the use of polymyxins and tigecycline during the study period, although the overall use is still relatively low (0.3% of overall DOT for systemic antibiotics). There are no comprehensive data about the use of “last-resort” antibiotics in US facilities, so direct comparisons over similar geographic regions are difficult. Two recent studies describing outbreaks of CR *Klebsiella pneumoniae* (Detroit 2009, Pittsburgh 2010) reported their ICU colistin use: 20.7 and 30.8 defined daily doses (DDDs) per 1000 patient-days respectively. [23], [24] Comparison with these data is difficult since they refer to outbreak settings. In addition caution has to be applied when comparing DDD to DOT, especially for drugs like polymyxins where there is significant uncertainty about the optimal dosing strategy. While the median antibiotic use density of polymyxin in VA ICUs was 2.1 DOT per 1,000 PD in ICUs that used at least one dose in 2010 there was one notable outlier at 57.3 DOT/1000 PD in FY 2010. In general, relatively few facilities, concentrated primarily in the Northeastern United States, contributed disproportionately to overall use.

Tigecycline is frequently used in conjunction with polymyxins and is often considered a “last-resort” antibiotic in its own right, but it seems to be used very differently. Compared to polymyxins, it appears to have been used more frequently in empiric treatment regimens and more often as a single agent. This is not surprising since tigecycline has been marketed for a relatively broad-range of indications, including community-acquired pneumonia and Gram-positive pathogens. We are not aware of any publicly available data on tigecycline use with which to compare; it will be interesting to follow its use in the VA given recent concerns about its effectiveness in severe infections. [13], [18].

The microbiology of Gram-negative isolates observed around the initiation of polymyxins demonstrated that this drug class was mainly used to treat resistant pathogens. There was a moderate correlation between polymyxin use in DOT and the number of drug resistant Gram-negative isolates over the study period. Although there is generally a two-way causal relationship between antibiotics and resistance, it seems unlikely that low-levels of polymyxin use greatly influence resistance to other antibiotic classes. We therefore surmise that the observed association between resistance and polymyxin use largely reflects the former driving the latter. Despite tigecycline being used more often than polymyxins, there were fewer resistant Gram-negative isolates associated with its use.

We were surprised to find that 30% of hospitalizations when polymyxins were used did not reveal any pathogens where susceptibility testing was performed. Possible explanations are that systemic polymyxins are often administered after long courses of broad spectrum antibiotics, or that some polymyxin use represents either an empiric escalation in patients failing therapy or empiric coverage of pathogens isolated more than a week prior to polymyxin initiation or from outside the VA. Non-fermenters were the most frequent pathogens and most isolates of *P. aeruginosa* and *Acinetobacter* spp. were MDR and CR; however, *Klebsiella* species were nearly as common as *P. aeruginosa* and also demonstrated frequent carbapenem and extensive-drug resistance. Even though data on strain typing or carbapenem resistance mechanisms were not available, KPC enzymes are known to be present in the VA system. [25], [26] Although tigecycline was sometimes used in association with *Pseudomonas* isolates, it should be noted that tigecycline is not active against this pathogen (idem for *Proteus* species) and in nearly all those case patient also received an anti-pseudomonal agent.

There is evidence of increasing carbapenem resistance throughout the United States. Thirty-eight states have reported CR Enterobacteriaceae (CRE) cases to the CDC in 2010 and some data indicate a dramatic rise in CRE in recent years. Given the sharing of patients between VAMCs, academic, and community hospitals, it is unlikely that VA trends will be independent from national trends.

Our study has several limitations. Although BCMA likely represents one of the most accurate ways to capture patient-level medication administration data and compliance with BCMA standards is regularly monitored within the VA system, we cannot be certain that all delivered doses were captured, which would underestimate antibiotic use. Similarly, since carbapenem resistance was difficult to identify in Enterobacteriaceae before revision of the Clinical and Laboratory Standards Institute (CLSI) carbapenem susceptibility breakpoints in mid-2010, and many facilities did not routinely test ertapenem in Enterobacteriaceae, we are likely to have underestimated carbapenem resistance in this family. [27] In addition, antibiotic susceptibility reporting is not standardized among VAMCs and some facilities routinely suppress antibiotic susceptibilities for certain organisms, potentially leading to an underestimation of carbapenem resistance. Multidrug resistance could also be biased simply from which susceptibilities laboratories chose to report. On the other hand, providers likely order cultures more frequently when they suspect resistance, thereby increasing the sampling of both colonizing and infecting organisms and biasing resistance estimates upward. Since the molecular mechanism of carbapenem resistance was not routinely determined, we were also unable to analyze the molecular epidemiology of carbapenem resistance in the VA system. Our approach to analyze microbiology culture within the 7th day before and 1st day after the first day of polymyxin or tigecycline use has not been formally validated. Electronic algorithms sometimes use, however, similar temporal rules. [28] Finally, the VA patient population may not be representative of other US hospitals and the findings of this study may thus not be generalizable to the entire United States.

To our knowledge this is the first study examining the use of “last-resort” antibiotics and the microbiology associated with their use in a large health-care system with detailed patient-level electronic data over several years. The total amount of polymyxin and tigecycline use is still relatively low in most VAMCs, but there has been an increase in the use of these drugs during the study period. Not surprisingly, our analysis shows polymyxin use is associated with MDR Gram-negative pathogens. The absence of antibiotics that are as effective and safe as the beta-lactams to treat infections with multi-resistant Gram-negative pathogens is a particular cause for concern. The Infectious Disease Society of America (IDSA) 10×‘20 initiative has called for the development of 10 new systemic antibacterial drugs by 2020 by facilitating the antibiotic approval pathway and creating incentives for drug manufacturers to develop new antibiotics. [29] This issue is also currently under review in the US congress (Generating Antibiotic Incentives Now (GAIN) Act). It remains to be seen if this goal can be met. In the meantime, strict infection control practice, combined with the judicious use of antibiotics in all settings (including the ambulatory care and veterinary sector), are our best weapons to address this threat. Electronic biosurveillance systems, more in-depth analyses about the characteristics and clinical outcomes of the patient group described in this study, and additional insight into the mechanisms of resistant pathogens transmission will be invaluable in this endeavor.
